# Blast-induced hearing impairment in rats is associated with structural and molecular changes of the inner ear

**DOI:** 10.1038/s41598-020-67389-5

**Published:** 2020-06-30

**Authors:** Ying Wang, Rodrigo T. Urioste, Yanling Wei, Donna M. Wilder, Peethambaran Arun, Venkatasivasaisujith Sajja, Irene D. Gist, Tracy S. Fitzgerald, Weise Chang, Matthew W. Kelley, Joseph B. Long

**Affiliations:** 1 Blast-Induced Neurotrauma Branch, Center for Military Psychiatry and Neuroscience, Walter Reed Army Institute of Research, Bethesda, MD USA; 2 Section on Developmental Neuroscience, National Institute on Deafness and Other Communication Disorders (NIDCD), Bethesda, MD USA; 3 Mouse Auditory Testing Core Facility, NIDCD, Bethesda, MD USA

**Keywords:** Trauma, Auditory system, Molecular neuroscience

## Abstract

Auditory dysfunction is the most prevalent injury associated with blast overpressure exposure (BOP) in Warfighters and civilians, yet little is known about the underlying pathophysiological mechanisms. To gain insights into these injuries, an advanced blast simulator was used to expose rats to BOP and assessments were made to identify structural and molecular changes in the middle/inner ears utilizing otoscopy, RNA sequencing (RNA-seq), and histopathological analysis. Deficits persisting up to 1 month after blast exposure were observed in the distortion product otoacoustic emissions (DPOAEs) and the auditory brainstem responses (ABRs) across the entire range of tested frequencies (4–40 kHz). During the recovery phase at sub-acute time points, low frequency (e.g. 4–8 kHz) hearing improved relatively earlier than for high frequency (e.g. 32–40 kHz). Perforation of tympanic membranes and middle ear hemorrhage were observed at 1 and 7 days, and were restored by day 28 post-blast. A total of 1,158 differentially expressed genes (DEGs) were significantly altered in the cochlea on day 1 (40% up-regulated and 60% down-regulated), whereas only 49 DEGs were identified on day 28 (63% up-regulated and 37% down-regulated). Seven common DEGs were identified at both days 1 and 28 following blast, and are associated with inner ear mechanotransduction, cytoskeletal reorganization, myelin development and axon survival. Further studies on altered gene expression in the blast-injured rat cochlea may provide insights into new therapeutic targets and approaches to prevent or treat similar cases of blast-induced auditory damage in human subjects.

## Introduction

Blast injuries have become prevalent in active duty military personnel due to increased exposure to improvised explosive devices and other explosives during combat operations^1,2^. The ears, eyes, lungs, and other air-filled or fluid-filled hollow organs are particularly sensitive to damage from the overpressure and/or under pressure of blast waves^3,4^. Auditory dysfunction, manifesting as hearing deficits and tinnitus, is the most prevalent neurosensory disability as a consequence of blast exposure. These disorders may persist for many years, as evidenced by long-term diagnoses of conductive or sensorineural hearing loss or mixed auditory deficits^5–11^. Clinical investigations indicate that blast exposures can damage the tympanic membrane, cartilage, ossicles, and muscles of the middle ear that together facilitate the transmission of sound to the inner ear^12,13^. Anatomical examination of inner ears from blast-exposed rodents reveals damage to the outer hair cells and to spiral ganglion neurons^9,14^ that accompany possible central processing disorders^15^.

Defects in auditory function can arise from multiple types and levels of injuries, each with significantly different potential recovery outcomes. Middle ear injuries can often be surgically repaired, but structural damage to the inner ear may have a limited ability to recover, due to death of specific associated neuronal cell types, such as hair cells and ganglion cells that can lead to permanent hearing deficits^16–18^. Within the inner ear, the hair cells are mechanosensitive to sound vibrations, as are the ganglion cells that innervate the hair cells and project their axons to the cochlear nuclei in the brain as the cochlear nerve a branch of the vestibulocochlear nerve. Like any sensor, there are a variety of other components that hair and ganglion cells and other neurons rely on for proper function. It is unclear whether the various sensory and supporting cells can be replaced, and which inflammatory responses have transient and/or permanent effects on auditory function.

A comprehensive understanding of the structural and molecular effects following blast shockwave injury is essential to develop the most appropriate therapies against resultant auditory deficits. Moreover, an appropriate animal model is critical for extending our understanding of ear damage repair and its limits. Our present study aimed to determine the molecular events in the inner ear along with structural changes in the middle ear that are associated with auditory functional deficits after blast exposure. We have employed RNA-sequencing (RNA-seq), quantitative real-time polymerase chain reaction (qRT-PCR), histopathology and functional assessments to provide a comprehensive profile of post-blast of auditory injury, repair, short- and long-term maladaptive responses.

## Results

### Tympanic membrane damages following blast exposure

**Figure 1:**
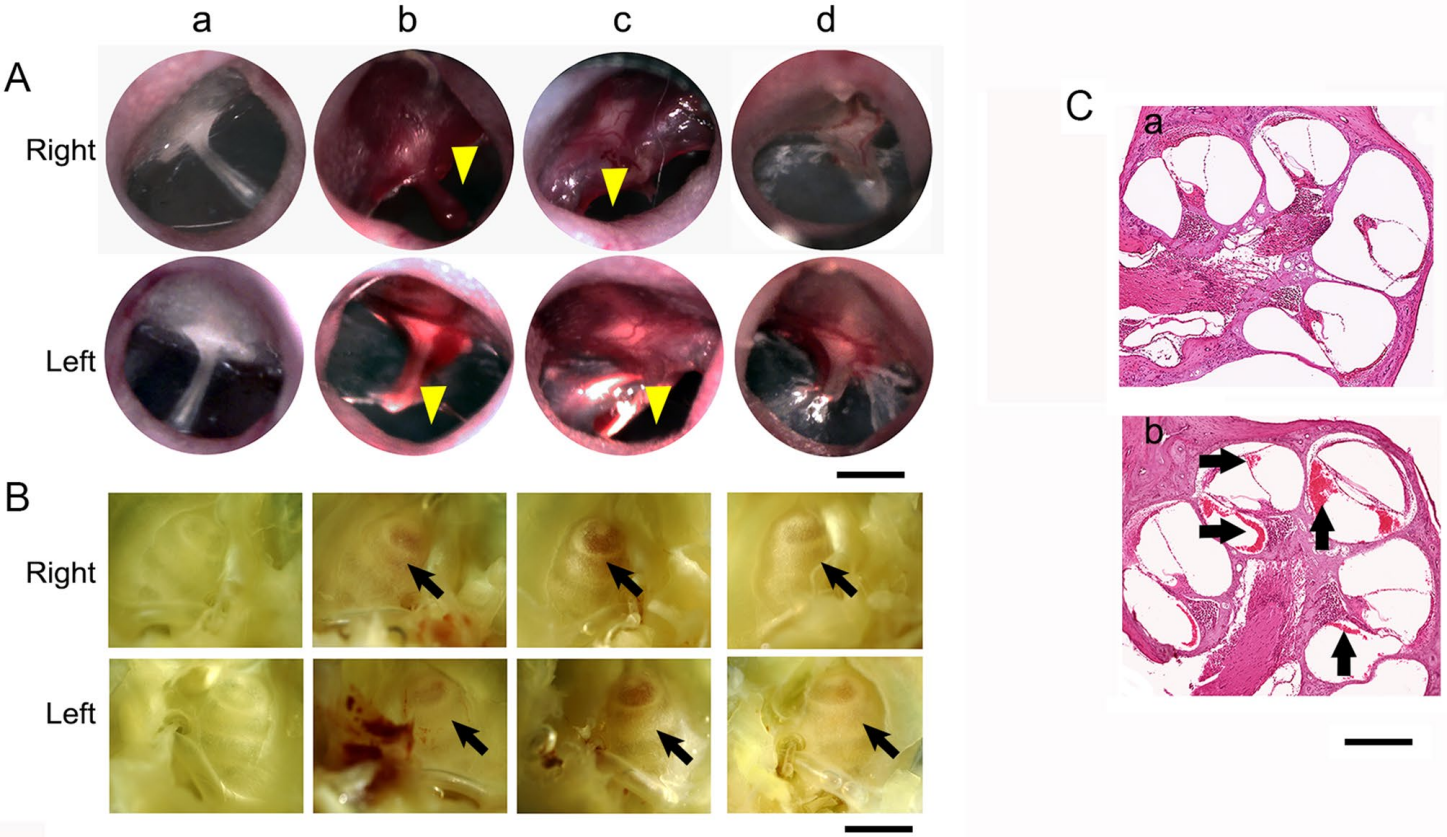
Blast exposure damage to ear structures. (**A**) Representative pictures of right and left tympanic membranes before (**a**) and at 1 (**b**), 7 (**c**), and 28 (**d**) days post blast exposure. Perforation (yellow arrow heads) and malleus scarring were evident. (**B**) Right and left middle ears of corresponding animals and profile of cochlea (arrow), a spiral-shaped cavity in the bony labyrinth shown above. (**C**) Representative paraffin-embedded cochlear cross-sections with H&E staining of sham (**a**) and at 7 days post injury (**b**). Blood cells (black arrows) were apparent shown in the scala tympani and vestibuli. The sections were 10 µm thick, scale bars A 1 mm, B 1 mm, and C 100 µm.

The integrity of tympanic membranes (TM) was assessed prior to and following blast exposures by otoscopy. As presented in Fig. 1A, each rat (n = 8) displayed a healthy TM prior to blast. Perforation of the TM was found immediately after blast exposure (data not shown). Damage of TM in both ears, middle ear hemorrhage, and swelling in the malleus region were apparent at 1 day (D1) and 7 days (D7) post injury. Healed TMs were evident in all rats at D28. After blast exposure, inner ear hemorrhage was not detected at 4 h, but was clearly visible at 1 and 7 days using a dissecting microscope under 20 × magnification (Fig. 1B). We further examined inner ears by assessing H&E stained cochlear cross sections (Fig. 1C) under a bright field microscope (200 × magnification). Exhibition of blood cells in the scala tympani and scala vestibule was discernable within the profile of inner ears, as shown in Fig. 1C-b.

### Blast-induced auditory functional deficits

Failure of response to acoustic stimuli has been considered as a basis for hearing loss. To determine the effects of blast-induced trauma on hearing, two cohorts of blast overpressure exposed rats (BOP) and sham control rats (SC) were evaluated with distortion-product otoacoustic emission (DPOAE) and auditory brainstem response (ABR). Prior to blast exposure (3–5 days), DPOAE and ABR were measured as the baseline (BL), and were then tested again on days 1, 7, 14, and 28 post-blast (D1, D7, D14 and D28) along with the measurements in sham controls at the same intervals.

**Figure 2:**
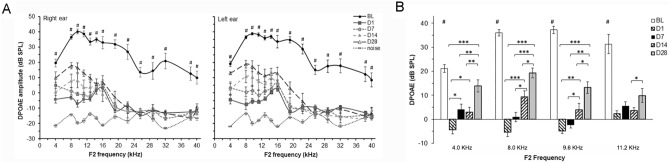
Impaired DPOAE amplitudes after blast exposure. (**A**) DPOAE responses of right and left ears for f2 = 4–40 kHz. Compared to baseline (solid line), DPOAE were significantly lower or absent across the observed frequency range at 1, 7, 14 and 28 days post-injury. There were no statistically significant differences between right and left ears after blast treatment. (**B**) DPOAE response comparisons after injury for low f2 frequencies at which some recovery of DPOAE was noted. Two-way ANOVA followed by Tukey’s multiple comparisons test; ^#^p < 0.01 with comparisons of BL measurements to all other time points, respectively at a specific frequency stimulus. *p < 0.05, **p < 0.01, ***p < 0.001, n = 8 each group.

DPOAE levels in response to two primary tones (L1, L2 = 65, 55 dB SPL and f2/f1 = 1.25) in the frequency range of f2 = 4–40 kHz were recorded. DPOAEs were present at all tested frequencies in all rats at baseline (BL), with the maximum values ranging from f2 = 4–10 kHz (Fig. 2A). The pattern of DPOAE responses recorded from the left ears following blast exposure was similar to that from the right ears which faced the shockwave. No significant differences in DPOAE amplitude levels were noted between the two ears of each animal. DPOAEs were absent at all measured frequencies at D1 and D7 post-blast. DPOAEs were present in a very narrow range from f2 = 8–9.6 kHz at D14, and were significantly higher than the DPOAE levels at D1 (p < 0.001) and D7 (p < 0.05) (Fig. 2B) post-injury. Compared to D14, DPOAE levels at D28 increased significantly (p < 0.05) in the range of f2 = 4–11.2 kHz, but decreased significantly (p < 0.0001) over all frequencies tested when compared to the BL. During the entire time frame of our investigation post-blast, no significant recovery was observed for DPOAE responses at frequencies from f2 = 12–40 kHz.

**Figure 3:**
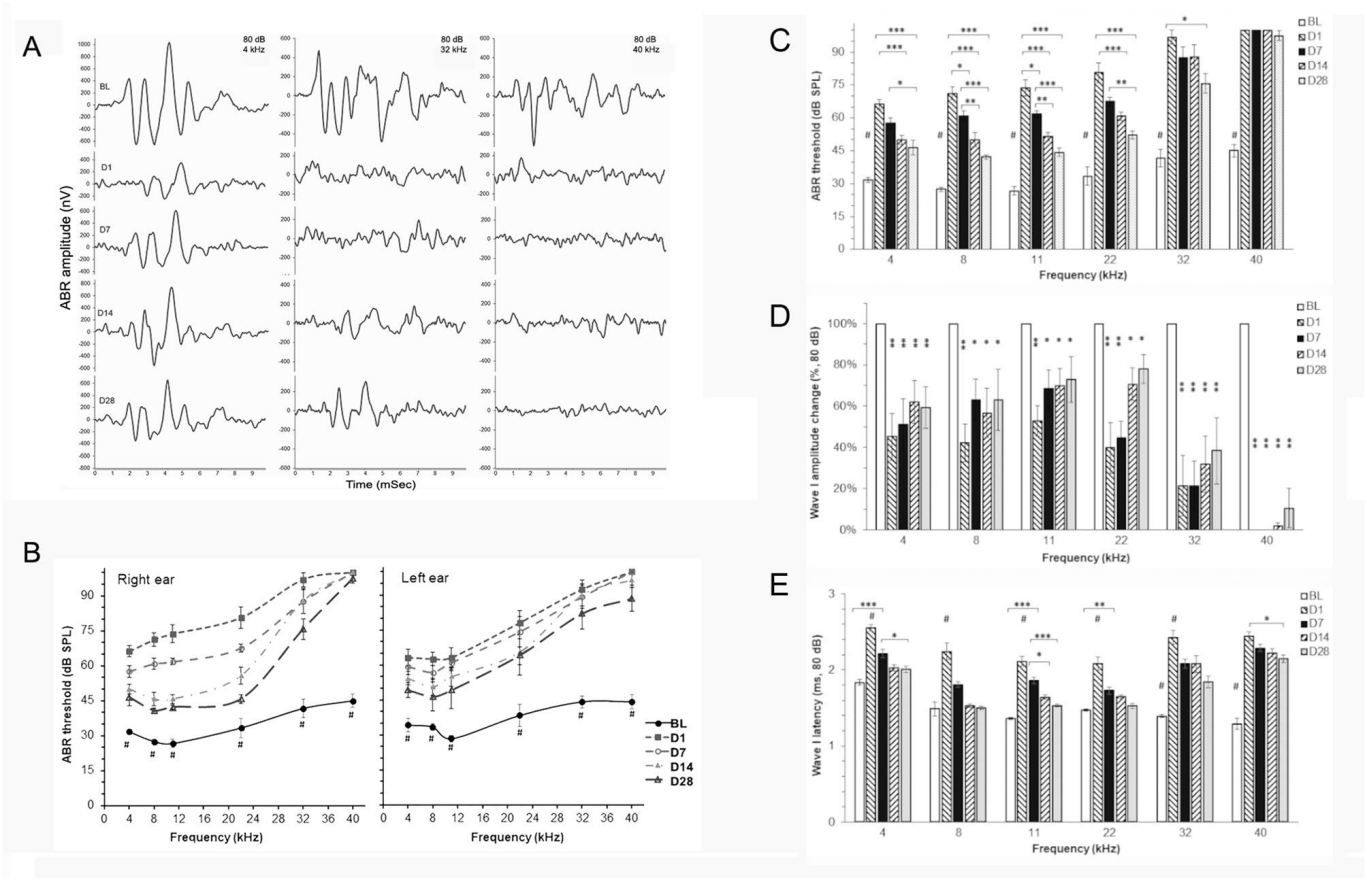
Impaired ABR thresholds after blast exposures. (**A**) Representative auditory brainstem responses during the first 10 ms after presentation of 80 dB SPL tone-burst at 4, 32 and 40 kHz. (**B**) ABR thresholds of right and left ears to tone burst stimuli from 4 to 40 kHz. ABR thresholds significantly increased at D1 across all test frequencies. Gradual recovery of ABR thresholds was noted in the low to mid frequencies (4–22 kHz) at D7, D14 and D28, although thresholds were still elevated relative to baseline (BL, solid line). (**C**) Right ear ABR threshold alterations following injury. Compared to acute injury at D1, ABR thresholds from 4 to 32 kHz stimuli partially recovered at D14 and D28, but were still significantly elevated compared to BL. Significant recovery was not observed at 40 kHz at all-time points tested after blast exposure. Percentage changes in right ear ABR wave I amplitude (**D**) and latency (**E**) following injury measured at 80 dB SPL. ABR wave I amplitude decreased and latency increased significantly at D1 compared to other time points. One-way ANOVA followed by Tukey’s multiple comparisons test, ^#^p < 0.01 for a group that was significantly different compared to another group; *p < 0.05, **p < 0.01, ***p < 0.0001, n = 8 each group.

All rats underwent ABR testing at 4, 8, 11, 22, 32 and 40 kHz tone bursts before and after blast exposure. ABR amplitudes declined dramatically at all test frequencies and peak response latencies increased significantly at D1 and D7 following blast exposure (Fig. 3A, D, E). Compared to the baseline, ABR thresholds (Fig. 3B, C) were significantly elevated in frequency stimuli ranging from 4 to 40 kHz at 1, 7, 14 and 28 days post-blast (two-way ANOVA). These changes showed no statistically significant differences between the right and left ears. Compared to D1, ABR thresholds at D14 and D28 post injury showed a significant reduction in the frequency range of 4–22 kHz. However, no or less recovery was detected at these two time points in ABR thresholds at 32 kHz and 40 kHz, respectively. Compared to its baseline, wave I amplitudes decreased greatly following blast exposure, particularly at D1 at all investigated frequencies (Fig. 3D). Over the subsequent 4 weeks, ABR wave I amplitudes showed gradually partial recovery at the frequency range 4–22 kHz, while no significant recovery was observed at 40 kHz. ABR wave I latencies were significantly prolonged at all tested frequencies on D1  compared to any other investigated time points (Fig. 3E). At D7, ABR wave I latencies were reduced significantly in the frequency ranges of 4–22 kHz relative to the measurements made on day 1, but the latencies were extended versus BL values. ABR wave I latencies at 40 kHz were significantly different among D1, D7 and D14 (one-way ANOVA).

### Alterations in gene transcription in cochlea of rats exposed to blast shockwaves

**Figure 4:**
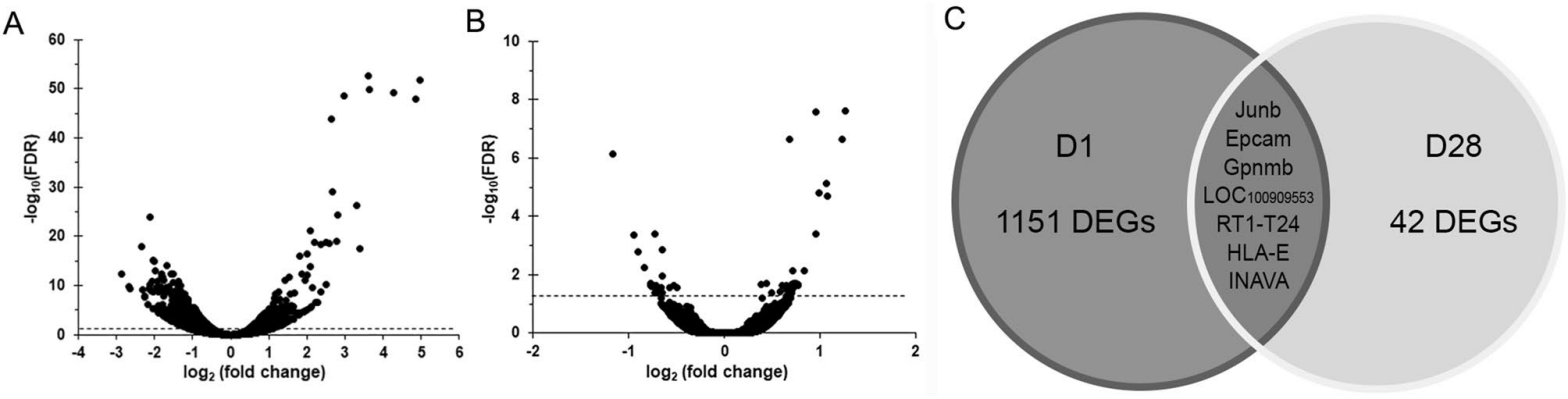
Alterations in differentially expressed genes (DEGs) after blast exposure. RNA-seq analysis from inner ear tissues demonstrated substantial differences in gene expression. (**A**) Volcano plots for the log2 (fold change) and − log10 (adjusted p value) of all DEGs in BOP1 vs SC1 at D1 post-blast, and (**B**) BOP2 vs SC2 at D28 post-blast, respectively. Statistical criteria for a gene to be considered differentially expressed were a fold-change ≥ 1.2 and an adjusted p value = 0.05. (**C**) Venn diagram depicting the distribution of the DEGs obtained from both acute (D1) and chronic (D28) phases. Seven genes are shared between D1 and D28.

To gain insight into what molecular changes occurred at acute and chronic phases post-blast, we performed RNA-seq on cochlea samples derived from blast exposed rats at 1 day (BOP1) and 28 days (BOP2) afterwards, as well as on samples from age-matched sham controls SC1 and SC2. The RNA samples were subjected to next-generation sequencing, which resulted in an average of 48.2 ± 2.1 million reads per sample. Perl script filtered reads were then mapped to the *Rattus norvegicus* genome. The overall RNA-seq results of the differentially expressed genes (DEGs) of BOP1 vs. SC1 (Fig. 4A) and BOP2 vs SC2 (Fig. 4B) are illustrated using volcano plots. According to a FDR-corrected p value of 0.05, 1,158 unique DEGs (3.98% of the total) were identified with 462 (40%) upregulated and 696 (60%) downregulated genes in BOP1 vs. SC1. In contrast, 49 DEGs (0.17% of the total) were identified with 31 (63.3%) upregulated and 18 (36.7%) downregulated genes in BOP2 vs. SC2. The distribution of the DEGs is represented as a Venn diagram (Fig. 4C). Seven DEGs, including known protein-coding genes as well as novel genes, were discovered at both the acute and chronic phases after injury. Based on a Log_2_ fold change cutoff of > 1.5, DEGs were sorted in a descending order of FDR values. The top 20 DEGs for each of up or down regulated protein-coding genes between BOP1 and SC1 are shown in Table 1. Compared to D1 post injury, the fold changes in DEGs were much less at D28 post injury. The top 15 DEGs for each of up or down regulated protein-coding genes between BOP2 and SC2 are shown in Table 2.

**Table 1:** Top 20 DEGs for identified up and down regulated genes at 1 day post-injury.

Up regulated genes				Down regulated genes			
Gene symbol	Fold change	pval	FDR	Gene symbol	Fold change	pval	FDR
Kng1l1	7.24	2.63E−55	1.90E−51	Fam163b	− 4.01	1.85E−12	1.06E−09
Kng2	6.34	1.07E−22	1.94E−19	Cabp7	− 3.63	7.74E−28	1.87E−24
Cxcl11	5.70	1.13E−13	8.44E−11	Cdh17	− 2.86	2.26E−07	3.56E−05
Lcn2	5.21	2.48E−55	1.90E−51	Chrna6	− 2.71	1.77E−18	2.40E−15
Lbp	4.08	3.48E−07	5.07E−05	Pcdh9	− 2.56	1.78E−09	4.89E−07
Timp1	4.03	4.66E−28	1.45E−24	Kcnc2	− 2.55	1.27E−17	1.53E−14
Wisp2	3.31	2.40E−30	4.08E−27	Pou4f1	− 2.44	4.66E−11	1.83E−08
Ifitm1	2.95	1.01E−11	4.68E−09	Lhfpl4	− 2.30	1.27E−12	7.50E−10
Ifitm3	2.82	2.37E−33	1.03E−29	Rph3a	− 2.27	1.46E−21	2.44E−18
Gpnmb	2.60	2.17E−22	2.08E−19	Shank1	− 2.26	5.47E−24	1.08E−20
RT1-T24	2.31	9.20E−14	7.39E−11	Kcnq4	− 2.23	5.27E−18	6.73E−15
Aqp1	2.08	2.55E−17	1.56E−14	Srgap3	− 2.23	3.45E−13	2.50E−10
Il17rb	2.04	2.57E−08	5.02E−06	Cpne4	− 2.23	7.13E−16	7.03E−13
Epcam	1.97	6.65E−10	2.03E−07	Ccdc68	− 2.23	1.32E−09	3.73E−07
Sfrp4	1.94	2.06E−14	8.51E−12	B3gat1	− 2.00	8.10E−13	5.49E−10
Mcm5	1.78	4.53E−06	4.24E−04	Map2	− 1.90	1.39E−10	4.93E−08
Ecel1	1.64	1.38E−05	5.71E−04	Apba1	− 1.80	1.08E−13	8.39E−11
PCOLCE2	1.58	9.31E−09	1.27E−06	Syn1	− 1.68	1.28E−09	3.71E−07
Enpp3	1.51	2.21E−07	3.53E−05	Syngr1	− 1.64	7.65E−11	2.82E−08
HLA-E	1.50	6.29E−10	1.06E−07	Cdk5r2	− 1.61	1.79E−11	8.07E−09

**Table 2:** Top 15 DEGs for identified up and down regulated genes at 28 days post-injury.

Up regulated genes				Down regulated genes			
Gene symbol	Fold change	pval	FDR	Gene symbol	Fold change	pval	FDR
Dsp	1.23	5.35E−11	2.30E−07	Slfn3	− 0.95	3.08E−07	4.37E−04
Clcnkb	1.08	9.95E−09	1.94E−05	Aqp4	− 0.90	1.33E−06	1.60E−03
Map3k6	0.99	6.71E−09	1.50E−05	Fabp7	− 0.84	5.15E−06	5.75E−03
Gpnmb	0.95	3.40E−12	2.65E−08	Ptprz1	− 0.77	4.42E−05	2.36E−02
Otog	0.95	2.46E−07	3.84E−04	Ermn	− 0.72	1.16E−04	4.13E−02
Kcnq1	0.83	7.22E−06	7.05E−03	Mlc1	− 0.72	2.25E−07	3.84E−04
Tbx1	0.76	4.99E−05	2.36E−02	Mog	− 0.72	8.04E−05	3.06E−02
Epcam	0.76	3.29E−05	2.18E−02	Psd2	− 0.71	1.34E−04	4.27E−02
Kcnn4	0.74	4.99E−05	2.36E−02	Gja1	− 0.69	7.71E−05	3.05E−02
Cdc42bpg	0.71	6.89E−06	7.05E−03	Chn2	− 0.66	6.65E−05	2.80E−02
Eps8l1	0.70	3.36E−05	2.18E−02	Tubb2b	− 0.65	1.31E−04	4.27E−02
Myh11	0.68	5.88E−11	2.30E−07	Plp1	− 0.65	1.02E−06	1.33E−03
Trim29	0.67	1.26E−04	4.27E−02	Lepr	− 0.58	6.56E−05	2.80E−02
Sik1	0.65	4.43E−05	2.36E−02	Ifi27	− 0.53	4.94E−05	2.36E−02
Bcam	0.44	2.57E−05	1.91E−02	Pygm	− 0.50	6.45E−05	2.80E−02

**Figure 5:**
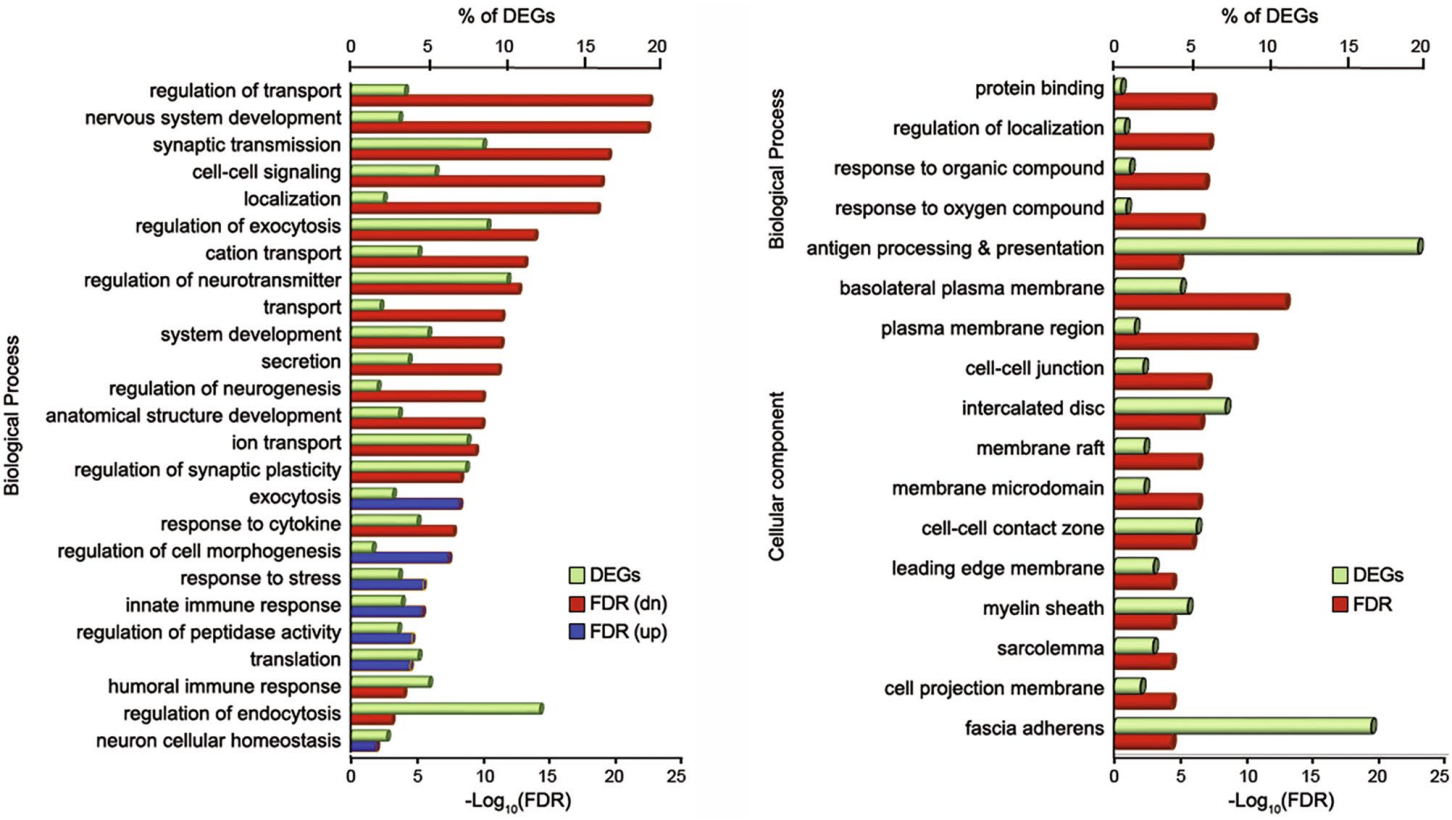
Gene Ontology enrichment analysis. (**A**) Top 25 biological processes for BOP1 vs. SC1 (order of the false discovery rate, FDR) were shown to be comprised of negatively regulated biological processes. (**B**) Major biological processes and cellular components identified for BOP2 vs. SC2. Percentage of DEGs belonging to each process are shown as green bars.

To examine the biological processes directly related to these genes, we performed DAVID gene ontology (GO) enrichment analysis on the DEGs data. The top 25 biological processes between BOP1 and SC1 are displayed in Fig. 5A as a percentage of total DEGs. The GO analysis demonstrated that the downregulated genes are involved mostly in biological functions, including neuro-cellular homeostasis (14.3%), regulation of neurotransmitter transport (11.8%), regulation of exocytosis (10.3%), synaptic transmission (10.0%), regulation of synaptic plasticity (8.8%), cell to cell signaling (6.4%), exocytosis (8.7%), secretion (5.9%), cation transport (5.1%), regulation of cell morphogenesis (5.0%), regulation of neurogenesis (4.4%), regulation of transport (4.1%), nervous system development (3.7%), ion transport (3.6%), localization (2.5%), system development (2.2%), and anatomical structure development (2.0%). The biological processes involved in the upregulated genes were entirely GO terms associated with defense functions, i.e., humoral immune response (5.1%), regulation of peptide activity (3.9%), innate immune response (3.6%), translation (3.6%), response to cytokines (3.2%), inflammatory response (2.7%) and response to stress (1.65%).

Compared to DEGs at day 1, significantly changed DEGs at day 28 were mainly enriched in molecular functions associated with protein binding within membranes. In the upregulated genes, GO clusters associated with cellular components for plasma membrane protein-interactions (Clcnka, Kcnn4, Kcnq10, Slc26a10, Eps8l1, Dsp, Epcam and Atp1a1), while the downregulated DEGs were mainly enriched in myelin sheath protein-interactions (Mog, Ermn and Plp1). The top altered GO terms associated with biological processes as a percentage of total DEGs were regulation of localization (1.0% ), response to oxygen-containing compounds (1.0%), response to organic cyclic compounds (1.0%), and antigen processing and presentation (18.2%) as shown in Fig. 5B. Identified DEGs in both acute and chronic phases of injury were associated with inner ear mechanotransduction, cytoskeletal reorganization, myelin development, and axon survival.

## Validation of RNA-seq results by qRT-PCR

The results from RNA-Seq indicated DEGs in both acute and chronic phases post-injury with a possible role in the cochlea’s response, and can be classified into two major groups: (1) genes involved in pathways that have a known role in molecular function of protein binding, cell to cell communication and ion channels, and (2) genes known to act as activation factors for the immune system. Although some genes have been well studied in the development of the middle and inner ear (Gsc) and cochlear hair cells (Atoh1, Pou4f3 and Myo7A), we found that their readcounts were 0–30 and thus failed here to be identified by RNA-seq analysis. We selected 13 genes from the RNA-seq that are known to be involved in ear development and auditory function maintenance (Atoh1, Aqp4, Cdh23, Coch, Gsc, Lhfpl5, Myo7a, Pou4f3, Prox1 and Otog), and the immune responses against injury (CCl21, Gpnmb and RT-1-T24) for confirmation of differential expression using quantitative real-time PCR.

**Figure 6:**
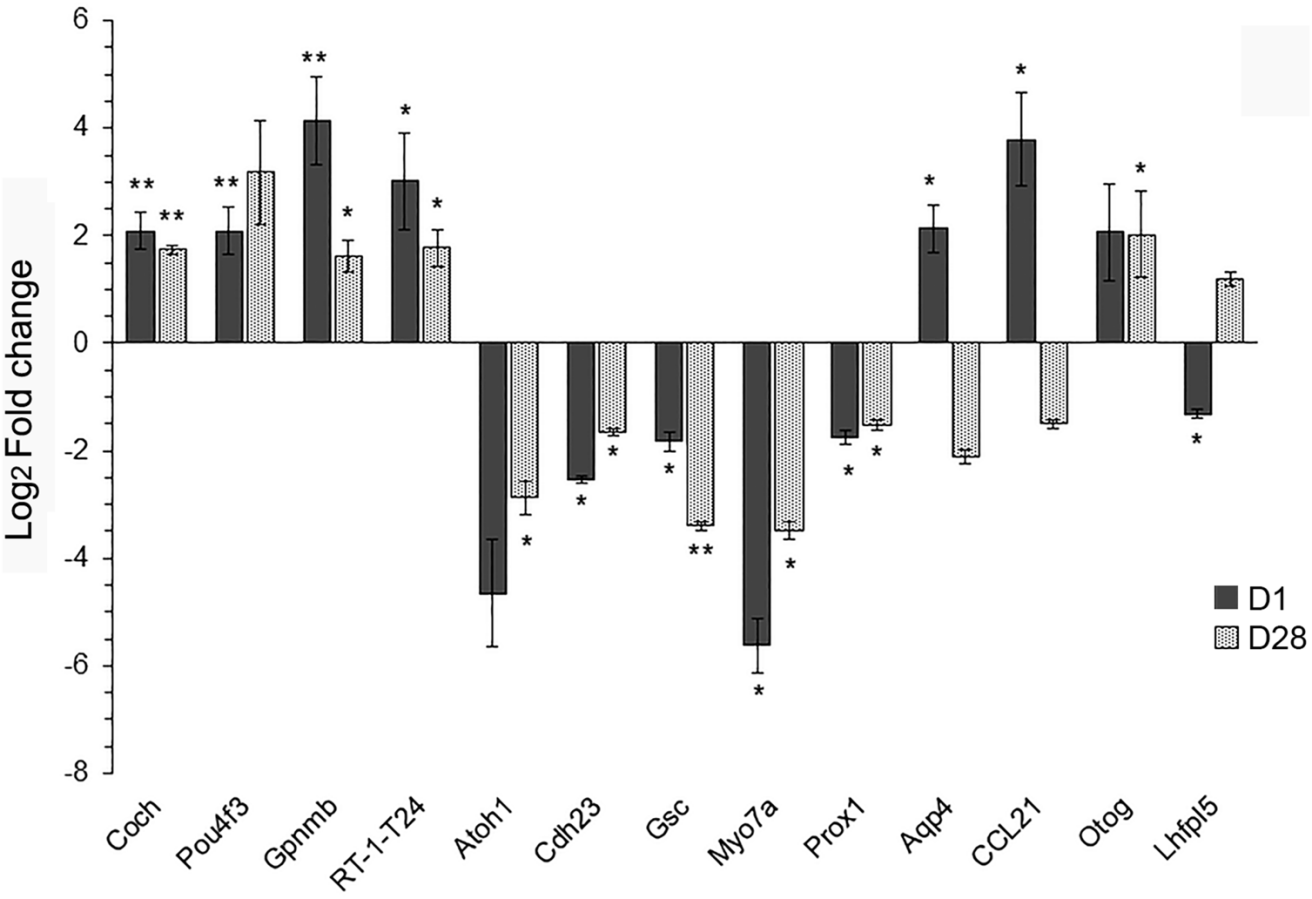
Validation of changes in gene expression by quantitative real-time PCR (qPCR). qPCR was performed to assess the mRNA levels of inner ear tissues. All primers were provided by QIAGEN. β-Actin served as an internal control. Data were expressed as mean ± SEM, D1 was compared with BOP1 (n = 5–6) and SC1 (n = 6), D28 was compared with BOP2 (n = 6–8) and SC2 (n = 8–10) using Student’s t test, *p < 0.05, **p < 0.01.

As shown in Fig. 6, Coch (p < 0.01 at D1, p < 0.005 at D28), Gpnmb (p < 0.01 at D1, p < 0.05 at D28) and RT-1-T24 (p < 0.05 at D1, p < 0.01 at D28) were significantly up-regulated, while Cdh23 (p < 0.005 at D1, p < 0.05 at D28), Gsc (p < 0.05 at D1, p < 0.005 at D28) and Prox1 (p < 0.01 at D1, p < 0.05 at D28) were significantly down-regulated at D1 (n = 5–6 rats for each group) and D28 (n = 6–9 rats for each group) post-injury, respectively. Aqp4 and CCL21 were significantly upregulated while Lhfpl5 was downregulated (p < 0.05) at D1. Otog was also found to be significantly upregulated at D28 (p < 0.05). Thus, the patterns of relative gene expression determined by qRT-PCR were the same as the data from the RNA-seq analyses.

## Discussion

The present study makes use of the ABS to characterize blast-induced auditory injury. In contrast to previous laboratory studies, the ABS produces shockwaves with Friedlander waveforms more representative of those created by explosives. The ABS incorporates an end wave eliminator and divergent transition section that eliminate artifacts found in commonly-used constant-diameter shock tubes, like high blast winds and plateau-top wave-forms^19^. Blast-induced acute auditory injuries are associated with an increased risk of chronic hearing loss. To elucidate the mechanisms of transition from acute to chronic injury, we identified ear structural changes and molecular alterations in rats exposed to tightly coupled repeated blast shockwaves^20^. Exposure to tightly coupled blasts is not uncommon either operationally or in military training and generates a more robust injury to the central nervous system which is relatively free of confounding polytrauma than is possible with a single higher amplitude blast wave exposure. In addition to improvised explosive device detonations, high overpressure weapon systems, such as artillery, can produce closely coupled consecutive overpressure-based insults. One practical advantage of using the tightly coupled repeated blast exposures is elimination of the need for additional exposure to isoflurane anesthesia, which is known to be neuroprotective.

Tympanic membrane perforation is one of the most frequent injuries of the peripheral auditory organ after blast exposure in military service members. Progressive hearing loss can be provoked by mechanical property alteration of the TM, which has been proposed to provide a readily-recognizable marker of blast conditions yielding concussive brain injury^21^. Others have reported that blast-induced TM perforation in mice is closely associated with the intensity of blast overpressure wave^14^. In our study, rats exposed to the tightly coupled double blast overpressure waves of ~ 16 psi (2 min interval) displayed a high incidence of TM perforation and middle ear hemorrhage and swelling, which occurred rapidly following the primary insults. The auditory damage in these rats persisted up to 7 days post exposure. Labyrinthine hemorrhage was not evident at 4 h, but became prominent from 1 day through 14 days post-injury. This implies the presence of a secondary injury from the blast is due to blood cell infiltration coming from middle ear hemorrhage. At day 28 post-blast, those middle ear structures that were damaged had healed with an increase in TM thickness. Overall, intensity-dependent blast-induced middle and inner ear damage was evident with no significant differences between the left and right ears and suggest that the use of suitable ear protection devices might effectively counteract the middle and inner ear injuries caused by blast exposure. This similarity of injuries in both ears was not unexpected. We believe that inner ear damage results from the increase in pressure which occurs relatively uniformly around the experimental subject during passage of the shock wave (and was recorded as static pressure) rather than as an impact of the shock wave with rat as dynamic pressure (e.g. blast wind) which would primarily affect the side of the rat facing the oncoming shock. Relative to constant diameter shock tubes, where dynamic pressure is artefactually exaggerated relative to free field measurements^19,22^, dynamic pressure is greatly reduced in the ABS and is a more fidelic recreation of free field blast flow conditions.

Our evaluation of the effects of blast exposure on auditory functions demonstrated acute and chronic impairments. DPOAE and ABR were measured before and after blast exposure at time points up to 28 days. Absence of DPOAEs was found immediately following blast exposure and up to 7 days post injury. DPOAE responses were detected within a limited frequency range from f2 = 8–9.6 kHz at 14 days and from f2 = 4–11.2 kHz at 28 days post exposure. At frequencies of f2 = 12 kHz and higher, DPOAEs were not detectable during the investigation period. Since DPOAEs arise from distortions in the outer hair cells (OHCs) mechanoelectrical response to two continuous tones, these results indicate that the OHCs were markedly impacted by the primary blast shockwave. DPOAEs also reflect the electro motile properties of OHCs^23,24^. Our data is consistent with previous findings by others of blast-induced OHCs loss in mice^14^.

ABR thresholds increased significantly over all tested frequencies from 4–40 kHz immediately after blast exposure and persisted for 28 days out. The earliest improvement in ABR threshold was noted at 7 days after injury in 8–11.2 kHz frequency range. By 28 days post-injury, the abnormally shifted ABR thresholds were recovered partially at 32 kHz, but no improvement was found at 40 kHz. Significant reduction of wave I amplitudes and prolonged wave I latencies were observed at all frequencies tested during the acute injury phase. As a measure of summed activity of neurons in the ascending auditory pathways, our ABR data provide information for our animal study regarding their auditory function and hearing sensitivity^25^. The effects of blast exposure on the left ears showed hearing loss with no statistical difference from that of right ears, although the rat’s right ear was facing the oncoming shockwaves. As noted above, both ears were exposed to comparable static pressures with similar injury outcomes. According to the distribution of sound frequency detection along the basilar membrane of cochlea, the stronger influence of blast exposure on high frequency hearing that we observed inferred that base of cochlea was more vulnerable to being injured. Nevertheless, it is important to note that for the level of functional recovery observed, we cannot distinguish to what extent it is due to healing of the tympanic membrane or cellular recovery in the cochlea. Since bone conduction ABRs were not performed, these contributions during functional recovery are also an open-ended question.

We also comprehensively analyzed molecular alterations in the inner ear post-blast using RNA-seq, which is the optimal method for large-scale transcription analyses. RNA-seq quantitates gene expression, enables novel gene discovery, and detects low abundancy genes^26,27^. RNA-seq profiling of the transcriptome of the whole cochlea yielded 29,050 genes that can be screened for alterations, which will help rapidly advance our understanding of molecular responses to blast-induced inner ear injuries. We observed significant changes in the degree of expression of numerous genes (DEGs) following blast exposure, when compared to sham controls. DEGs were subsequently classified according to their biological processes and molecular functions, especially those related to supporting auditory and other neuronal functions. Notable in our results is that enriched GO clusters at one day post-blast comprised largely negative regulation of biological process, in comparison to GO clusters at 28 days post exposure which were characterized by positively regulated pathways. This is consistent with an acute phase injury response followed by a chronic phase recovery response. The top categories of DEGs at the acute phase involved regulation of cellular localization, cation channel activity, regulation of transport, nervous system development, neurotransmitter release and cell–cell signaling. During the chronic phase the DEGs were more limited to cell–cell junction, plasma membrane formation, protein binding, and antigen processing and presentation.

Implications of the DEGs may be limited in scope, however, due to known drawbacks of RNA-seq, such as the technical challenges in library construction and bioinformatics noise. Therefore, validation of our RNA-seq results was necessary. Loss of connectivity of the ear is a major burden underlying blast-induced hearing dysfunction. We performed qRT-PCR analysis on Gsc mRNA levels in cochlea, which were DEGs that showed substantial changes following blast exposure. The Gsc protein acts as a transcription factor and is essential for tympanic ring development^28^ and inner ear development^29,30^. It is also a crucial regulator of mesodermal patterning in mammals and has specific functions in neural crest cell derivatives^31^. Gsc is the most abundantly expressed homeobox gene in the vertebrate organizer^32,33^. Deletion of the Gsc in mice^34^ or loss of Gsc function in humans^31^ lead to multiple craniofacial defects. Experimental mis-expression of Gsc strongly promotes epithelial-to-mesenchymal transition and enhances metastasis^35,36^. Gsc plays a role in prechordal cells to promote migration and to inhibit convergent extension. It is also expressed in the tissues that undergo tissue remodeling at later stages, such as neural crest-derived mesenchymal tissues^37^. The qRT-PCR analysis showed that expression of Gsc was down-regulated significantly at days 1 and 28 after blast exposure and suggests that intracochlear injections using therapies which can modulate the levels/activity of Gsc in the cochlea may be an effective therapeutic strategy against blast-induced hearing impairments.

The key components of the acoustic system include sensory hair cells (HCs). The genes of Atoh1 and pou4f3 are well known for the development of HCs, although no genes have been discovered for which expression is limited only to inner ear HCs. Both of them are transcription factors. Expression of Atoh1 is limited primarily to embryonic precursors of both supporting cells and HCs, with later limitation to developing HCs^38^. The pou4f3 gene is required for the late differentiation of HCs^39,40^, which may be a direct target for Atoh1 regulation^41^. Moreover, HCs of inner ear mainly contain Myosin VIIA (Myo7a) protein. Mutation in the Myo7a gene is also thought to be involved with autosomal recessive non-syndromic hearing impairment. Any variation in the Myo7A gene is responsible for about 50% of different types of Usher syndromes^42,43^. However, mRNA of Atoh1 was undetectable and the readcounts of pou4f3 and Myo7a were very low in the RNA-seq results. Our qRT-PCR data revealed that Atoh1 and Myo7a are significantly downregulated, but pou4f3 was significantly upregulated at days 1 and 28 after injury. Mechanosensory transduction by HCs depends on a protein complex at the tips of shorter stereocilia associated with mechanoelectrical transduction channels activated by tip links in the hair bundle^44^. Lhfpl5 is a tetraspan membrane protein that distributes throughout the hair bundle in the inner ear. It modulates transducer channel conductance^45^. Cdh23, one of two atypical cadherins that form the fine filaments is essential for the mechanotransduction^46^. Significant decreases in mRNA levels of Lhfpl5 and Cdh23 at 1 day post-blast were confirmed by qRT-PCR. Blast-induced downregulation of Cdh23 was found at 28 days post-blast.

Our studies of acute and chronic immune responses in the cochlea after blast exposure revealed that cytokines (e.g. Ccl) were upregulated only at the acute phase, while Gpnmb, INAVA, HLA-E and RT1-T24 were upregulated in both acute and chronic phases. The Gpnmb gene, which is strongly associated with lipid-laden macrophages^47,48^, plays an important role in the regulation of inflammation responses^49^. Upregulation of Gpnmb gene was found by others to occur in the brains of rats following stroke^50^, and cerebral ischemia–reperfusion injury^51^. It also is implicated in the impairment of T-cell activation^52^, as well as being thought to induce autophagy during specific stress conditions^53^.

### Conclusion and prospective

In summary, we comprehensively analyzed effects of blast injury in rats on cochlear function using ABRs and DPOAEs, and on mRNA expression levels using PCR analysis. Cochlear signaling response to a sound stimulus was found to be markedly impaired by one day post-blast. Likewise, many genes were shown to be differentially expressed at one day post-blast and some of them were abnormally prolonged through a month out. The results revealed that blast overpressure injury to the ear leads to morphological and transcriptional changes that affect auditory function and govern regenerative processes. Our findings using a high-fidelity blast simulation model have elucidated pathways as well as specific genes that may be involved in blast-induced hearing loss which might enable the development of suitable strategies for both prevention and cure of blast-induced hearing impairments. With the high prevalence of blast injuries characterized by both short- and long-term auditory deficits, we can now better understand the associated pathological processes in the inner ear. Further study of altered gene expression may yield additional fruitful insights into new methods for treating or preventing blast-induced auditory deficits, by identifying targets for the actions of experimental drugs and those already approved by the FDA.

## Methods

### Animals and blast injury

**Figure 7:**
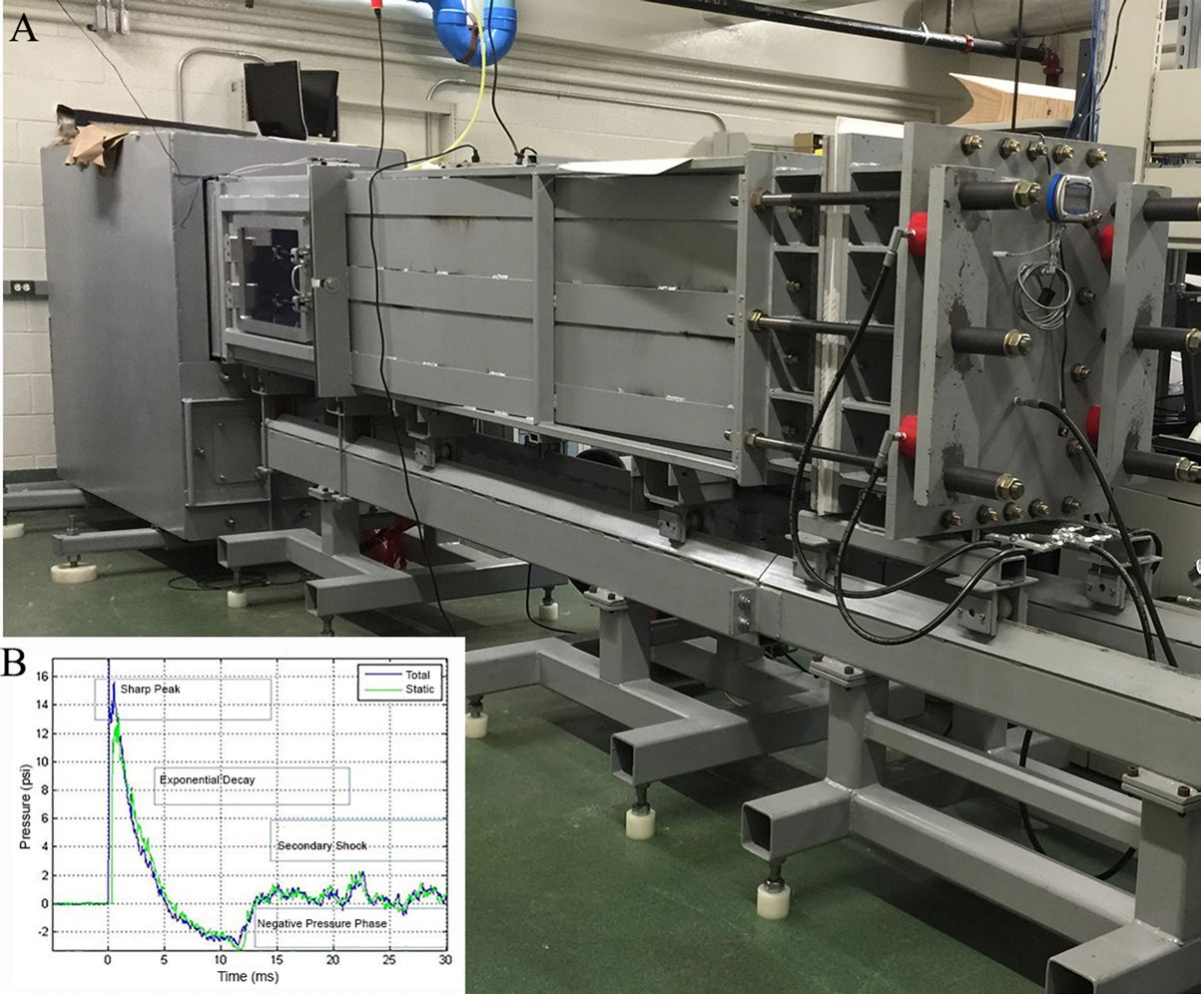
(**A**) Advanced blast simulator, (**B**) pressure recording from tip/side gauges.

All animal experiments were conducted in accordance with the Animal Welfare Act and other federal statutes and regulations relating to animals and experiments involving animals and adhered to principles stated in the Guide for the Care and Use of Laboratory Animals with an Institutional Animal Care and Use Committee approved protocol. Male Sprague Dawley rats, 320–350 g, obtained from the Charles River Laboratory were separated randomly into two experimental groups: tightly coupled double blast exposures (BOP) and sham control (SC). For tightly coupled repeated blast exposures, the animals were exposed to two 16 psi blast overpressure waves separated by 2 min. Exposure to repeated shock waves in this manner is not uncommon either operationally or in military training and generates a more robust insult to the central nervous system that is relatively free of complicating polytrauma than is possible with a single exposure to a higher amplitude waveform^20,54^. The blast overpressure (peak static pressure of 16 psi and ~ 4 ms positive phase duration) was generated by rupture of a VALMEX (Mehler Technologies) membrane mounted in an advanced blast simulator (ABS), which consists of a 0.5 ft. long compression chamber that is separated by the membrane from a 21 ft. long transition/expansion test section (Fig. 7)^19,20,55^. The compression chamber is rapidly pressurized with air causing the membrane to rupture at a pressure that is dependent upon its thickness, yielding a supersonic shockwave that impacts the experimental subject. Immediately after anesthetization by exposure to 4% isoflurane gas in an induction chamber for 6 min (O_2_ flow rate 1.5 L/min), rats were secured in the ABS in a prone position with the right side towards the oncoming shockwave. BOP experimental subjects received two tightly coupled blast shockwaves with a 2 min interval, during which time additional isoflurane anesthesia was delivered. SC animals were included in all individual experiments and were handled in the same fashion, including being subjected to isoflurane anesthesia, but without exposure to blast wave.

### Auditory function testing

Distortion-product otoacoustic emission (DPOAE) and auditory brainstem response (ABR) are commonly used for hearing assessment^56^. For these evaluations, rats were anesthetized by intraperitoneal injection (IP) of a mixture of Ketamine (50 mg/kg) and DEXDOMITOR (0.3 mg/kg). DPOAE and ABR were measured using Tucker-Davis Technologies (TDT, Alachua, FL, USA) hardware (RZ6 Multi I/O processor, MF-1 speakers) and software (BioSigRz, v.5.7.2) as described^57^. For ABR measurement, sub-dermal needle electrodes were placed at the vertex of the skull and beneath each pinna, with the non-test ear serving as the ground. During the procedure, animals were placed on a heating pad, set to 37 °C, in a sound-proof booth. ABR thresholds were determined by presenting Blackman-gated tone-burst stimuli of 3-ms duration at six frequencies (4, 8, 11.2, 22.4, 32 and 40 kHz). Supra-threshold stimulus intensities were initially decreased in 10-dB steps, followed by 5-dB steps at lower intensities to establish the final thresholds. When no ABR waveform was detectable at the highest intensity level of 90 dB SPL, the threshold was considered to be 95 dB SPL for subsequent analysis. DPOAE signals were obtained in response to two primary tones with frequency ratio f2/f1 = 1.25 and intensity levels f1 = 65 and f2 = 55 dB SPL which was varied in one-fifth-octave steps from 4 to 40 kHz. The output in dB V were converted to dB SPL offline using an Excel spreadsheet that based on the ER-10B + microphone’s calibration voltage.

### Tympanic membrane evaluation

The integrity of the rat tympanic membranes was evaluated using a Digital Macroview Otoscope (Welch Allyn, Skaneateles Falls, NY, USA).

### Middle/inner ear dissection and histopathology

Rats were deeply anesthetized with 4% isoflurane for 8 min and then euthanized by exsanguination by transcranial perfusion with 0.9% saline, which was then followed by buffered 4% paraformaldehyde (PFA). The temporal bone was removed and immersed in 4% PFA for 2 days, after breaking open the tympanic bullae. The middle ear photographs were taken under a dissecting microscope (Olympus SZX2-ZB16). After softening for 5 days in 0.2 M EDTA in 0.1 M phosphate buffer (pH 7.0) solution for decalcification, the inner ear was embedded in paraffin blocks. Sections (10 µm in thickness) were cut and stained with hematoxylin and eosin (H&E) to evaluate histological morphology. Images of cochlear cross-sections were taken by a bright field microscope (Olympus BX-63).

### Cochlea dissection and RNA isolation

The temporal bones of euthanized rats were immediately covered with powdered dry ice and stored at − 80 °C. Cochlear tissues including the organ of Corti and ganglion cells were dissected and pooled from two ears of the same rat; pooled samples were used for total RNA extraction using RNeasy Plus Mini Kit (Qiagen, Germanton, MD, USA) according to the manufacturer’s instructions. RNA concentration and quality were determined by NanoDrop ND-2000 (ThermoFisher, USA). The RNA integrity number (RIN) was measured using Agilent 2100 Bioanalyzer system (Agilent Technologies, USA). The RNA samples (RIN > 7) were further processed for RNA-seq or qRT-PCR analyses.

### RNA-Seq and analysis

Library constructs and RNA sequencing were performed by Novogene (Novogene Corporation Inc., Sacramento, CA, USA). Briefly, the library constructs for cochlear total RNA were generated using a TruSeq Stranded mRNA Sample Prep Kit (Illumina, USA) following the manufacturer’s protocol. Library concentration was quantified using a Qubit 2.0 fluorimeter (ThermoFisher, USA), and then diluted to 1 ng/µl before checking the RNA insert size on an Agilent 2100 Bioanalyzer and quantifying it to a greater accuracy by qPCR (library activity > 2 nM). Libraries were applied to an Illumina HiSeq 2,500 system (Illumina USA). Raw data of fastq format were first processed through an in-house Perl script to increase the quality of the data (Novogene Corporation). HTSeq v0.6.1 was used to count the reads mapped to each gene. Differential expression analysis between two groups was performed using DESeq2 R package. The resulting p values were adjusted using the Benjamin and Hochberg’s approach for controlling the false discovery rate (FDR). Genes with an adjusted p value < 0.05 found by DESeq2 were assigned as differentially expressed gene group. Gene ontology (GO) enrichment analysis of differentially expressed genes was implemented by the GOseq R package, in which gene length bias was corrected. GO terms with corrected p value less than 0.05 were considered significantly enriched by differentially expressed genes.

### Quantitative RT-PCR

All primers and reagents were obtained from QIAGEN. Briefly, cDNA was converted from 300 ng of high-quality cochlear total RNA for each sample using a RT^2^ Easy First Strand kit. Each sample was analyzed three times. Quantitative PCR was performed on the cDNA by combining with a RT^2^ SYBR Green qPCR Mastermix reagent and followed by amplification on an Applied Biosystems 7500 Fast Real-Time PCR System (ThermoFisher, USA). No template control samples were included as negative controls. Relative expression was normalized to the level of the housekeeping gene β-actin and calculated using 2^−ΔΔCt^ method.

### Statistical analysis

Statistical analyses were performed using GraphPad Prism 6 software (GraphPad Software Inc., San Diego, CA, USA). Data were analyzed using Student’s t test or repeated measures ANOVA. For multiple comparisons, following ANOVA, significant differences among treatment groups were identified using Tukey’s multiple comparison tests. Differences were considered to be significant at the level *p* < 0.05 and values are expressed as the mean ± standard error.

## Data Availability

All data are available on request.
